# Allosteric regulation of the nickel-responsive NikR transcription factor from *Helicobacter pylori*

**DOI:** 10.1074/jbc.RA120.015459

**Published:** 2020-11-22

**Authors:** Karina A. Baksh, Dmitry Pichugin, Robert Scott Prosser, Deborah B. Zamble

**Affiliations:** 1 Department of Biochemistry, University of Toronto, Toronto, Ontario, Canada; 2 Department of Chemistry, University of Toronto, Toronto, Ontario, Canada

**Keywords:** allosteric regulation, metalloprotein, transcription factor, nickel, DNA-binding protein, metal homeostasis, bacterial transcription, structure–function, nuclear magnetic resonance (NMR), CD, circular dichroism, DBD, DNA-binding domain, DTNB, 5,5'-dithiobis-(2-nitrobenzoic acid), EcNikR, *E.coli* NikR, 5F-Trp-HpNikR, 5-fluorotryptophan-labeled *H. pylori* NikR, HpNikR, *H. pylori* NikR, IPTG, isopropyl-β-D-thiogalactopyranoside, PhNikR, *P. horikoshii* NikR, MBD, metal-binding domain

## Abstract

Nickel is essential for the survival of the pathogenic bacteria *Helicobacter pylori* in the fluctuating pH of the human stomach. Due to its inherent toxicity and limited availability, nickel homeostasis is maintained through a network of pathways that are coordinated by the nickel-responsive transcription factor NikR. Nickel binding to *H. pylori* NikR (HpNikR) induces an allosteric response favoring a conformation that can bind specific DNA motifs, thereby serving to either activate or repress transcription of specific genes involved in nickel homeostasis and acid adaptation. Here, we examine how nickel induces this response using ^19^F-NMR, which reveals conformational and dynamic changes associated with nickel-activated DNA complex formation. HpNikR adopts an equilibrium between an *open* state and DNA-binding competent states regardless of nickel binding, but a higher level of dynamics is observed in the absence of metal. Nickel binding shifts the equilibrium toward the binding-competent states and decreases the mobility of the DNA-binding domains. The nickel-bound protein is then able to adopt a single conformation upon binding a target DNA promoter. Zinc, which does not promote high-affinity DNA binding, is unable to induce the same allosteric response as nickel. We propose that the allosteric mechanism of nickel-activated DNA binding by HpNikR is driven by conformational selection.

*Helicobacter pylori* is a gram-negative pathogenic bacterium that infects the human stomach, causing various gastrointestinal diseases including peptic ulcers, chronic gastritis, and gastric cancer (1, 2, 3). The bacteria thrive at neutral pH and have robust acid acclimation mechanisms to withstand the fluctuating pH in the stomach, which can drop as low as 1.8 (4, 5, 6). A protein central to the acid adaptation response of *H. pylori* is the nickel enzyme urease, which hydrolyzes urea from the host into ammonia to neutralize the intracellular pH and the local microenvironment (5, 6, 7, 8). Urease requires 24 nickel ions to complete the active enzyme and can account for up to 10% of the total cellular protein content, making nickel an essential nutrient for *H. pylori* (7, 8, 9). Nickel is also a cofactor for [NiFe]-hydrogenase, which allows *H. pylori* to use hydrogen gas produced by other gut bacteria as an energy source (10, 11).

The limited availability and inherent toxicity of nickel mean that maintaining desirable levels of this metal necessitates an extensive network of nickel acquisition, storage, delivery, and efflux pathways (9, 12, 13, 14, 15). In many organisms that utilize nickel, these systems are coordinated by the nickel-responsive transcription factor NikR, which is referred to as a metalloregulator or metal-sensor protein (16, 17, 18, 19). In *H. pylori*, NikR (HpNikR) senses the bioavailability of nickel and subsequently activates or represses transcription of a variety of genes encoding nickel homeostasis proteins and acid adaptation factors, including the urease enzyme precursor proteins (17, 18, 20, 21, 22, 23). HpNikR binds an oligonucleotide recognition sequence in the promoter of target genes that consists of two half sites that are a pseudo-symmetric palindrome separated by a spacer (24, 25, 26, 27). Binding nickel allosterically activates complex formation with this DNA recognition sequence, resulting in transcriptional regulation (16, 28, 29, 30, 31). HpNikR-regulated promoters have a weak consensus sequence (24, 26) and are roughly divided into two tiers based on whether they are bound by HpNikR with affinities in the nanomolar or micromolar range (25). The mechanism by which nickel is able to activate this DNA-binding response is currently unclear.

Crystal structures of HpNikR (32, 33, 34, 35, 36), and its homologs from *Escherichia coli* (EcNikR) (37, 38, 39), and *Pyrococcus horikoshii* (PhNikR) (40) reveal a homotetramer, with a central C-terminal metal-binding domain (MBD) connected by apparently flexible linkers to two flanking N-terminal DNA-binding domains (DBDs) that have a ribbon–helix–helix fold (Fig. 1*A*). Four identical, and conserved, square planar His_3_Cys nickel sites per tetramer were observed in the structures of EcNikR (37, 38, 39, 41) and PhNikR (40), and in solution NikR proteins bind nickel with high affinity (28, 42, 43, 44). The same sites are present in structures of HpNikR (35), although additional coordination sites have also been observed under different conditions (32, 34, 36). Crystal structures have also provided snapshots of several conformational states of NikR—*open*, *trans*, and *cis*—defined by the positions of the DBDs relative to the MBD (Fig. 1*B*) (32, 37, 38, 40). HpNikR is believed to adopt an ensemble represented by the *open*, *trans*, and *cis* conformers, where the presence of nickel and target DNA sequences likely influence both the conformational equilibrium and the ability of the DBDs to undergo the rigid-body movement that enables exchange between states. In the DNA-bound EcNikR crystal structure, the protein was observed in the “*cis*” conformation, in which both DBDs make contact with the recognition sequence along a single interface (38). However, crystal structures of NikR without DNA reveal the *open* and *trans* conformers, regardless of nickel binding, suggesting that nickel alone does not induce a conformational shift or large changes in tertiary structure (32, 37, 39, 40). In EcNikR, nickel binding has been shown to induce short-range effects in the MBD to allosterically activate DNA binding, but there is no evidence to support the same type of mechanism in HpNikR (16, 17). Instead, allostery in HpNikR is believed to be driven by changes in dynamics, specifically the mobility of the DBDs (16, 45, 46, 47).

**Figure 1:**
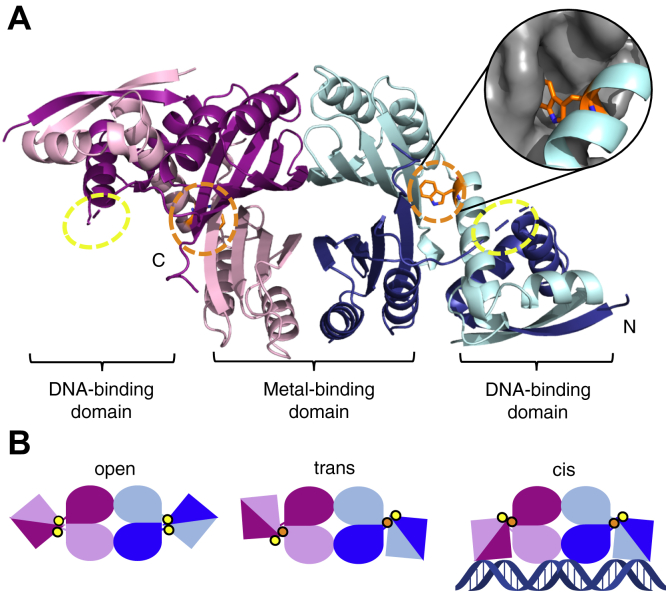
**Crystal structure of apo-HpNikR (PDB: 2CA9) and tetrameric model states.***A,* Crystal structure of apo-HpNikR showing two protomers in different shades of *pink* and two in different shades of *blue*. Trp54 (*orange*) is only resolved in two protomers, here represented in *light pink* and *light blue*. Inset: the two crystallographically resolved Trp54 residues are surrounded by a pocket created in the MBD (the MBD is shown as a *gray surface*). The unresolved Trp54 are presumed to be solvent-exposed, and their approximate positions are indicated with a *yellow circle*. *B*, Schematic representations of observed NikR conformations defined by the positions of the DBDs relative to the MBD. *Circles* represent the approximate locations of Trp54, with those expected to be solvent-exposed in *yellow* and those expected to be buried in *orange*.

In this work, we sought to determine the mechanism by which nickel binding activates DNA binding by HpNikR. As is the case for other metalloregulators (16, 48, 49), HpNikR is capable of weakly interacting with DNA upon binding metals other than nickel—most notably, zinc (30). However, only nickel elicits the correct allosteric response required for tight DNA binding (30). Using ^19^F-NMR, we examined changes in the conformational states and dynamics of HpNikR upon binding nickel or zinc and upon complexation with a well-characterized DNA target. Our studies reveal that HpNikR adopts a fast equilibrium between an *open* state and more DNA-binding competent *cis*/*trans* configurations. Addition of nickel shifts the equilibrium toward the *cis*/*trans* conformers and increases their lifetime. Thus, nickel allosterically biases the ensemble toward long-lived DNA-binding competent states in a manner consistent with conformational selection.

## Results

The HpNikR tetramer possesses four tryptophan residues (Trp54), which are located in α-helix 2 on the DBDs at the interface with the MBD (Fig. 1*A*) (32, 34). By biosynthetically labeling the protein with 5-fluorotryptophan, Trp54 serves as a useful ^19^F-NMR reporter of the *open*, *trans*, and *cis* equilibria as a function of metal and DNA. ^19^F-NMR chemical shifts are sensitive to subtle differences in solvent exposure and electrostatic environments (50, 51). In the full-length apo- and nickel-bound HpNikR crystal structures, the protein is observed in the *trans* state where only two tryptophans are resolved and appear to be buried in a pocket in the MBD (Fig. 1*A*) (32, 34). The remaining two tryptophans that are not resolved are thought to be in a solvent-exposed region of the protein, as indicated in Figure 1. Trp54 is conserved in PhNikR, and in the crystal structures of Ni(II)-PhNikR in the *trans* state, all four are resolved and located in environments similar to those in the HpNikR structures, where two appear more buried while the other two are more solvent-exposed (40).

The *cis* state has only been observed in the crystal structure of Ni(II)-EcNikR in complex with DNA (38). Although Trp54 is not conserved in EcNikR, published sequence alignments of NikR homologs reveal that it corresponds to Thr45 (17, 32, 40), all four of which are resolved in the structure of the Ni(II)–EcNikR–DNA complex (38). Two of the Thr45 residues appear to be solvent-exposed, while the other two are more buried, similar to the environments observed for the tryptophan residues in the *trans* state of HpNikR and PhNikR. Therefore, the environments of the tryptophan residues at the MBD/DBD interface are likely to be similar when the protein is in either the *cis* or *trans* state—as depicted by the yellow and orange circles in Figure 1*B*—making it possible that these two states could be indistinguishable in the ^19^F-NMR spectra.

The crystallographic *open* state of apo-PhNikR reveals that all four tryptophans are in seemingly equivalent positions relative to the MBD (40). We therefore anticipate that when HpNikR is in the *open* state, the four tryptophans would also be in nearly equivalent environments (Fig. 1*B*). Thus, the spectra originating from the fluorotryptophan label at the MBD/DBD interface should be sensitive to conformational changes (*cis*/*trans*/*open*), local conformer-specific dynamics, as well as DNA binding (51). Biosynthetic ^19^F-labeling of HpNikR with 5-fluorotryptophan does not perturb the secondary structure of the protein, as well as its nickel- or DNA-binding activities (Fig. S1), and this construct was therefore used to probe the impact of metal ions and DNA on HpNikR.

### HpNikR samples multiple conformations in the presence and absence of nickel

**Figure 2:**
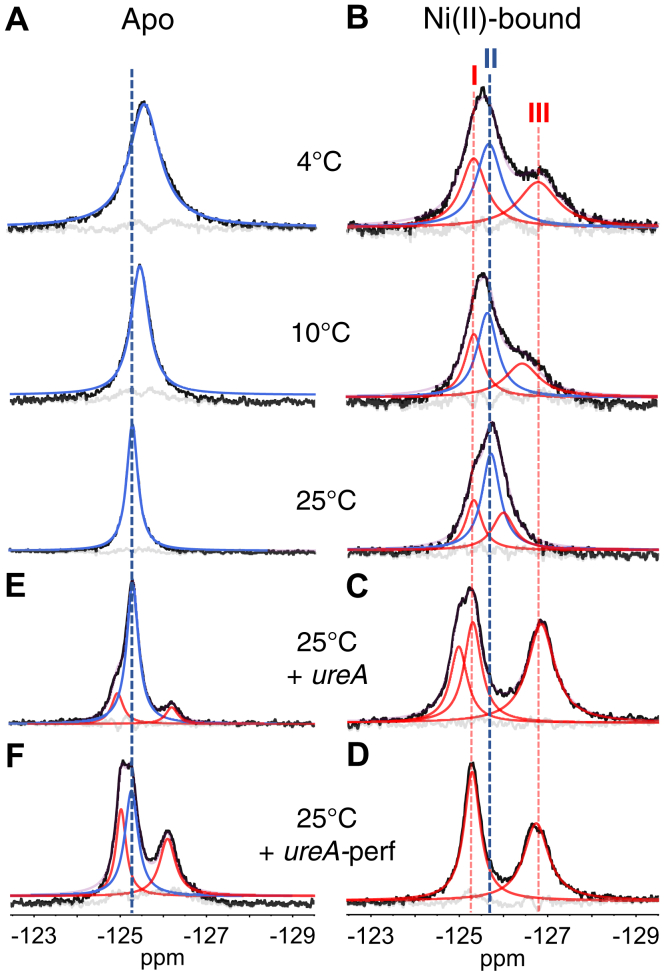
**Deconvolved**^**19**^**F-NMR spectra of 5F-Trp-HpNikR.** The *black lines* correspond to the original spectra, and the fit peaks are colored according to their assignments. *A*, the fit peak for the apo-protein (*blue*) is believed to represent a weighted average of the states. *B*, in the nickel-bound protein, I and III (*red*) correspond to the *cis*/*trans* states, and II (*blue*) corresponds to the *open* state. *C* and *D*, show the nickel-bound protein with the *ureA* promoter and a modified version of this promoter named *ureA*–perf, respectively. In both cases, no *open*-state signature can be detected, and the protein adopts an asymmetric or symmetric *cis* state, respectively. *E* and *F*, show the apo-protein with the *ureA* and *ureA*–perf promoter, respectively. Fit peaks in *red* correspond to DNA-bound populations. The *faint purple lines* indicate the sums of the fits, and the *gray lines* are the residual errors associated with the fits.

The ^19^F-NMR spectrum of apo-5F-Trp-HpNikR collected at 4 °C reveals a single peak, indicating that all four tryptophans might be in an equivalent environment, as expected for the *open* state (Fig. 2*A*). However, *T*_*2*_ relaxation experiments of the apo-protein reveal a modest degree of inhomogeneous broadening, suggesting there are subtle differences between the fluorotryptophan reporters in the tetramer, which are likely masked by local dynamics and by fast sampling of conformations due to the mobility of the DBDs relative to the MBD (Fig. S2). The narrowing of the peak with increasing temperature is consistent with the interpretation that the apo-protein undergoes fast conformational exchange and that the individual tryptophan residues likely undergo fast local reorientations.

The addition of nickel to 5F-Trp-HpNikR results in two distinct resonances at lower temperatures (Fig. 2*B*). Deconvolving the spectrum of nickel-bound 5F-Trp-HpNikR acquired at 10 °C into two resonances suggests that linewidths of the downfield and upfield resonances are on the order of 550 Hz and 840 Hz, respectively (Table S1). However, these values are not consistent with the results of *T*_*2*_ relaxation studies, which instead suggest that the homogeneous contributions to the downfield and upfield resonances are 300 Hz and 400 Hz, respectively (Fig. S3*A*). For this reason, we deconvolved the spectra of nickel-bound 5F-Trp-HpNikR into three resonances (I, II, and II), as shown in Figure 2, resulting in fitted linewidths of 410, 440, and 630 Hz, respectively (Table S1), which are much closer to the linewidths estimated by *T*_*2*_ relaxation experiments (Fig. S3*B*, Table S1).

In general, downfield resonances in ^19^F-NMR spectra correspond to more buried ^19^F probes, whereas upfield resonances indicate greater solvent exposure (51, 52). The ratio of the areas of resonances I and III—designated in red in Figure 2*B*—are consistently 1:1, implying that two tryptophan residues in the nickel-bound HpNikR tetramer are situated in a more buried environment (*i.e.*, resonance I) while the other two tryptophan residues are more solvent-exposed (resonance III), which is in accordance with the expected environments associated with either the *cis* or *trans* states. The intermediate resonance (II) is more apo-like and likely corresponds to the *open* state where all four tryptophans are in equivalent positions relative to the MBD. HpNikR binds nickel stoichiometrically with high affinity, and nickel titrations indicate that saturation occurs around 0.9 equivalents of nickel per monomer (Fig. S1*B*) (28, 42, 43, 44). The protein was therefore incubated with 1.2 equivalents of nickel per monomer for these experiments to ensure that it is nickel-loaded.

The temperature series of the ^19^F-NMR spectra of both apo- and Ni(II)-5F-Trp-HpNikR exhibit coalescence of the single or multiple resonances, respectively, with increasing temperature, suggesting that there is local dynamics and conformational exchange regardless of nickel binding. There is also a clear downfield shift of the resonance in the apo series with increasing temperature (Fig. 2*A*). One possible explanation for this temperature dependent shift is that the protein is in fast exchange between the *open*, *cis*, and *trans* conformers in the absence of nickel; at higher temperatures, there is further coalescence of the resonances associated with those states such that the weighted average causes an overall downfield shift. To provide additional information and aid in the assignment of the resonances, ^19^F-NMR spectra were also acquired upon addition of DNA.

### Binding the *ureA* promoter stabilizes Ni(II)-HpNikR in an asymmetric cis conformation

The crystal structure of the Ni(II)–EcNikR–DNA complex revealed that the *cis* conformation is DNA-binding competent (38). While the crystal structures of apo- and Ni(II)-HpNikR reveal little structural differences (32), the ^19^F-NMR spectra indicate that HpNikR adopts a dynamic equilibrium between the *open* state and the *cis* and/or *trans* states in the absence of DNA. To examine the conformation of HpNikR in the DNA-bound complex, ^19^F-NMR spectra were recorded upon the addition of a 32 bp oligonucleotide containing the *ureA* promoter. This sequence is a well-known target of HpNikR and is bound by the nickel-loaded protein with a low nanomolar *K*_d_ (Fig. S1*D*) (21, 25, 28, 29, 30, 47). The downfield and upfield resonances in the ^19^F-NMR spectrum of Ni(II)-HpNikR bound to this promoter have equal areas, although the most downfield resonance appears slightly asymmetric and is best deconvolved into two overlapping resonances (Fig. 2*C*). Thus, the deconvolved spectrum exhibits three resonances with an area ratio of 1:1:2, which likely correspond to two unique buried tryptophans and two equivalent solvent-exposed tryptophans in the tetramer. These environments are consistent with those expected for either the *cis* or *trans* states, which appear to be indistinguishable by ^19^F-NMR. However, HpNikR is thought to bind DNA in the *cis* conformation since both DBDs would then engage both half sites of the recognition sequence, as is observed in the Ni(II)–EcNikR–DNA crystal structure (38). Therefore, in this case, it is likely that these resonances correspond to the cis state. The large separation between the downfield and upfield resonances is likely due to the protein adopting a single conformation when bound to DNA, resulting in the absence of conformational exchange with the open state and therefore the absence of coalescence; this does not rule out very slow exchange. No change in the spectrum was observed upon adding more DNA, and no coalescence was detected with increasing temperature (Fig. S4), supporting the conclusion that the DNA-bound Ni(II)–protein is stabilized in a single conformation.

As mentioned above, the presence of two overlapping downfield resonances in the spectrum of the Ni(II)–5F–Trp–HpNikR–DNA complex suggests that each of the two DBDs is slightly inequivalent when bound to the *ureA* promoter. This result is consistent with a previous study in which a nuclease assay revealed asymmetric cleavage patterns of the *ureA* promoter when bound to HpNikR (53). The authors believed that the asymmetry was due to the DBDs being in distinct conformations when bound to this promoter (53). A likely factor for this binding mode is the pseudo-palindromic half sites of the HpNikR recognition sequence. A thymine found on only one half site of the recognition sequence was previously found to be critical for tight DNA binding and is conserved among the promoters bound by HpNikR with high affinity, such as the *ureA* promoter (27). To investigate whether there is asymmetric binding to this promoter due to its nonsymmetric recognition sequence, ^19^F-NMR was performed with a mutated version of the *ureA* promoter composed of a perfect palindrome with both half sites containing the critical thymine (named *ureA*-perf (25)). Fluorescence anisotropy measurements indicate that HpNikR binds the 32 bp *ureA*-perf promoter with a similar affinity as the *ureA* promoter (Fig. S5). The ^19^F-NMR spectrum of Ni(II)–5F–Trp–HpNikR with the *ureA*–perf sequence revealed a single downfield peak with an area equal to that of the upfield peak, as shown in Figure 2*D*. These results indicate that upon binding the *ureA* recognition sequence, nickel-bound 5F-Trp-HpNikR becomes stabilized in a slightly asymmetric *cis* conformation. This asymmetry of the *cis* conformation is not observed in the Ni(II)–EcNikR–DNA crystal structure, which is not surprising because EcNikR binds a symmetric recognition sequence (38, 43).

HpNikR is also capable of binding the *ureA* and *ureA*–perf promoters in the absence of nickel, but with an affinity that is orders of magnitude weaker than that of the nickel-bound protein (Fig. S5). Accordingly, the spectra of the apo-protein incubated with the *ureA* or *ureA*–perf promoter, as shown in Figure 2, *E* and *F*, are dominated by one major peak (blue), similar to the spectrum without DNA, making it likely that this peak corresponds to the unbound protein. The spectra also exhibit a DNA-bound signature, characterized by both downfield and upfield resonances in a 1:1 ratio (red), which are less separated than those seen in the nickel-bound species, suggesting that the apo-protein adopts a weakly populated DNA-bound complex that is different than the DNA complex formed by the nickel-bound protein. These results are consistent with the fluorescence anisotropy results that show HpNikR is capable of weakly binding DNA in the absence of nickel, but nickel is required for high-affinity binding (Fig. S5).

### Ni(II)–HpNikR samples the DNA-binding competent conformer in the absence of DNA

The resonances produced by the DNA-bound conformer align well with resonances I and III in the spectra of the nickel-bound protein without DNA; this is evident in the spectrum collected at 4 °C where a greater separation of the resonances is observed (Fig. 2, *B–D*). These data support the interpretation that resonances I and III correspond to a DNA bound-like *cis* conformer that is sampled by the nickel-loaded protein in the absence of DNA. However, due to the inability to distinguish between the *cis* and *trans* states in the spectra, resonances I and III could also correspond to the protein adopting the *trans* state and are therefore designated as the *cis*/*trans* states when DNA is absent. It is possible that the *trans* state serves as an intermediate in the DNA-binding process wherein one DBD engages one half site of the *ureA* promoter, resulting in an allosteric response in which the protein interconverts to the cis state and binds the second DNA half site. Resonance II, which is the apo-like signature, has been designated as the open state where all four Trp54 are likely to be in nearly equivalent environments.

**Figure 3:**
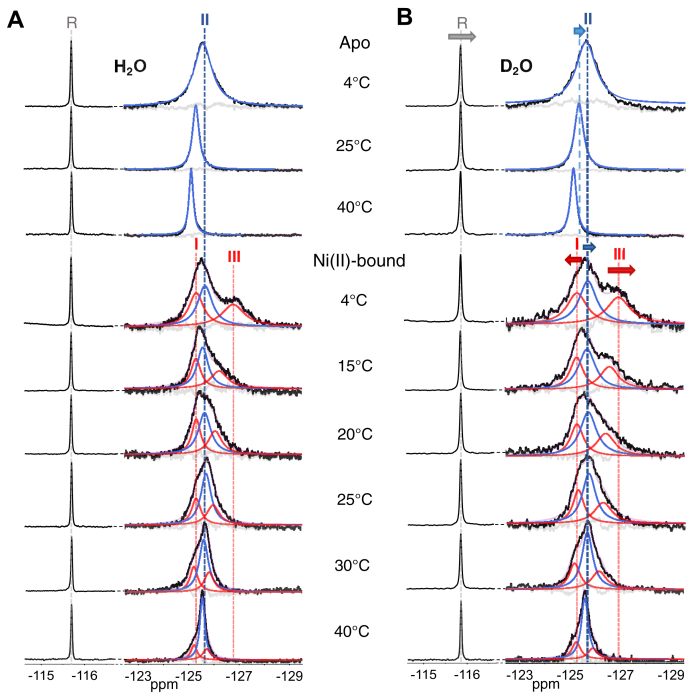
**Deconvolved**^**19**^**F-NMR spectra of apo- and Ni(II)-5F-Trp-HpNikR in (*A*) 10% D**_**2**_**O and (*B*) ∼90% D**_**2**_**O.** The colors of fit peaks are the same as described for Figure 2. The *arrows* in (*B*) indicate the direction of the shifts of the resonances in ∼90% D_2_O, with *larger arrows* indicating a larger chemical shift difference compared to the spectra shown in (*A*). The reference peak to which the spectra are aligned is indicated by “R” and exhibits a consistent upfield shift in ∼90% D_2_O.

To validate the assignments of the resonances, additional spectra were collected at identical temperatures but in the presence of ∼90% D_2_O, generating a solvent-induced isotopic shift effect (Fig. 3) (51, 52). Typically, the substitution of D_2_O for H_2_O results in an upfield “solvent isotope shift” of as much as 0.25 ppm for fully solvent-exposed probes (51, 52). The apo-protein signature is observed to shift upfield, consistent with a state in which all four tryptophan residues in the tetramer are under fast exchange and are solvent-exposed (Fig. 3, Fig. S6*A*). Resonance II for Ni(II)–5F–Trp–HpNikR also exhibits an upfield shift (Fig. S6*B*), consistent with the expected solvent exposure of the tryptophans when the protein is in the *open* state. Resonances I and III for the nickel-bound protein are observed to shift downfield and upfield, respectively, supporting their designation as the *cis*/*trans* states where two tryptophan residues in the tetramer are in a more buried environment (resonance I) while the other two are more solvent-exposed (resonance III) (Fig. S6, *B* and *C*). The slightly larger separation of resonances I and III suggests that exchange dynamics are reduced, indicating that the *cis*/*trans* states are longer lived in the nickel-bound protein. D_2_O is known to increase the stability of water-mediated hydrogen bond networks (51, 54, 55), which could play a role in increasing the lifetime of the *cis*/*trans* states. A careful comparison of the temperature series in H_2_O and D_2_O also reveals an effective temperature shift. For instance, the spectrum acquired at 20 °C in D_2_O resembles the nonshifted spectrum at 15 °C, which is in accordance with the interpretation that D_2_O reinforces water-mediated hydrogen bonded networks in the protein, thereby reducing exchange dynamics (51, 54, 55).

To learn more about the thermodynamics of the interconversion between the open and *cis*/*trans* states by the nickel-bound protein, a Van’t Hoff analysis of the temperature dependence of the state populations in H_2_O and D_2_O was conducted (Fig. S7). The $\Delta H_{open,cis/trans}^{{}^{\circ}}$ was calculated to be −20.7 kJ/mol and −18.9 kJ/mol in H_2_O and D_2_O, respectively, and the $\Delta S_{open,cis/trans}^{{}^{\circ}}$ was calculated to be 70.2 J/mol K and 63.5 J/mol K. These values suggest the transition from *open* to *cis*/*trans* is both enthalpically and entropically driven. The positive entropy terms could be a result of both increased configurational dynamics and a loss of bound water, both of which may play a role in DNA recognition and binding, but future work will be required to investigate this possibility. Altogether, the ^19^F-NMR results of Ni(II)–5F–Trp–HpNikR in D_2_O and H_2_O at different temperatures are consistent with the conclusion that the nickel-bound protein samples the DNA-binding competent *cis/trans* conformer in the absence of DNA and that the *cis/trans* conformer is characterized by the asymmetric doublet consisting of resonances I and III.

### Nickel binding slows down DBD mobility and shifts the conformational equilibrium to promote DNA binding

A comparison of the temperature series of spectra shown in Figures 2, *A* and *B*, and 3*A* associated with the apo- and nickel-bound protein reveals a prominent resonance (blue), which we attribute to the *open* state for Ni(II)–5F–Trp–HpNikR (resonance II). In the spectra of the nickel-bound protein, this resonance only slightly shifts downfield with increasing temperature, while the resonance in the spectra of the apo-protein exhibits a larger downfield shift. This difference may result from the apo-protein undergoing rapid exchange between the *open* and *cis/trans* conformers. As mentioned above, higher temperatures likely bring about further coalescence of the *open* and *cis/trans* signatures in the apo-protein, such that the weighted average results in an overall downfield shift with increasing temperature. In contrast, the DNA bound-like *cis/trans* signatures (I and III) are clearly separated for Ni(II)–5F–Trp–HpNikR, but begin to coalesce in the absence of DNA, as shown in Figure 2*B* at 25 °C. The resonances associated with the DNA-bound state of the apo-protein are also largely reduced in the absence of DNA, indicating increased exchange dynamics. These data support the conclusion that nickel increases the DNA binding competent *cis*/*trans* fraction of conformers, while also increasing the *cis*/*trans* lifetime.

Taken together, the above results suggest that both nickel-bound and apo-HpNikR adopt an equilibrium between an *open* and a *cis*/*trans* state that is capable of binding DNA. These results are also consistent with previous atomistic molecular dynamics studies and small-angle X-ray scattering (SAXS) experiments that demonstrated that nickel binding does not induce a conformational shift to the *cis* state (33, 36, 45). Instead, nickel appears to reduce the DBD mobility and allosterically shift the conformational equilibrium to the DNA-binding competent *cis*/*trans* states, which have concomitantly increased lifetimes. The results are consistent with a conformational selection mechanism, which posits that while the protein is able to sample the key functional states, the addition of nickel greatly increases the lifetime of the *cis*/*trans* conformers needed for DNA binding and transcriptional regulation.

### Zinc does not induce the same allosteric response as nickel

Metalloregulators generally bind to metal ions other than their cognate metal, but only the cognate metal allosterically stimulates the complete DNA-binding response (16, 48, 49). For example, DNA binding by HpNikR can be activated upon loading with cobalt or zinc, but with a weaker affinity than nickel (30). To examine whether a noncognate metal has the same allosteric impact on HpNikR as nickel, ^19^F-NMR experiments were performed with zinc. A previous study determined using isothermal titration calorimetry (ITC) that HpNikR binds 6 Zn(II) ions per tetramer in two high affinity sites (*n*_1_ = 2, *n*_2_ = 4) with a *K*_d1_ of 6 ± 3 nM and a *K*_d2_ of 90 ± 20 nM. Therefore, for the ^19^F-NMR and fluorescence anisotropy experiments, HpNikR was incubated with 6.4 equivalents of ZnSO_4_ per tetramer for a slight excess of zinc to ensure that the protein is zinc-saturated.

**Figure 4:**
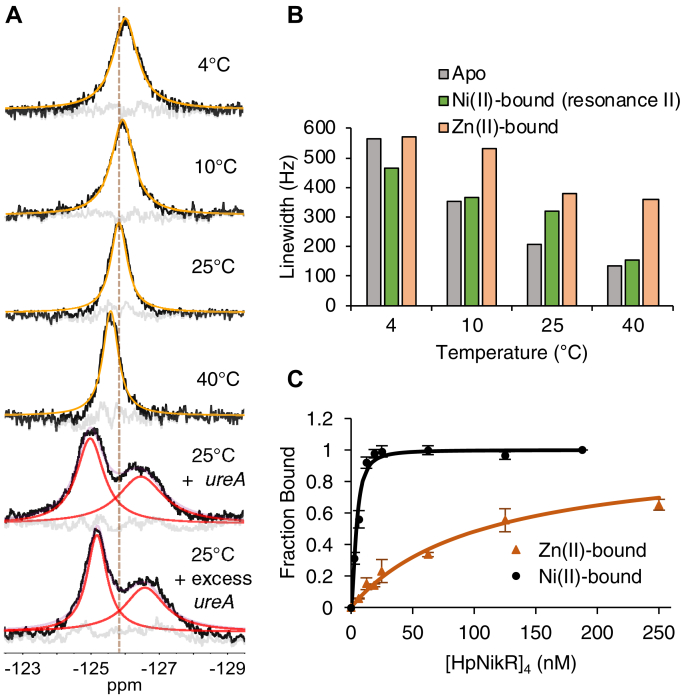
**Deconvolved**^**19**^**F-NMR spectra of zinc-bound 5F-Trp-HpNikR with and without the *ureA* promoter, in addition to linewidth analysis and DNA binding isotherms.***A,*^19^F NMR spectra with deconvolutions. The fitted resonance shown between 4 °C and 40 °C is believed to result from averaging between *open* and *cis*/*trans* conformers, as discussed in the text. Fitted peaks in *red* are assigned to the DNA-bound *cis*/*trans* conformer. *B*, Temperature dependence of the linewidth of resonances of Zn(II)-bound, apo, and resonance II of nickel-bound 5F-Trp-HpNikR. *C*, DNA binding of Ni(II)- and Zn(II)–HpNikR. Fluorescence anisotropy experiments were performed with 5 nM of the *ureA*-F promoter in 3 mM MgSO_4_, 20 mM Tris, 100 mM NaCl, pH 7.6. The data points represent the average derived from the preparation of three samples at each protein concentration, and the error bars represent ± one standard deviation. The data from each replicate were fit to the Hill equation. The average DNA-binding affinities were determined to be 4.5 ± 0.6 nM, n = 1.8 ± 0.1 and 107 ± 19 nM, n = 1.0 ± 0.05 for the nickel- and zinc-bound tetrameric proteins, respectively.

In contrast to the nickel-bound spectra shown in Figure 2*B*, the spectra of Zn(II)–5F–Trp–HpNikR reveal a single resonance, which both shifts downfield and decreases in linewidth with increasing temperature (Fig. 4, *A* and *B*). Notably, this trend is reminiscent of the temperature-dependent spectra of apo–5F–Trp–HpNikR, shown in Figure 2*A*. We can therefore conclude that zinc binding is insufficient to establish a long-lived *cis*/*trans* signature in the absence of DNA and that the single observed resonance is the result of fast conformational exchange between an *open* state and a *cis*/*trans* state. The larger linewidth for the Zn(II)-bound protein compared to the apo-protein suggests that zinc binding comparatively increases the lifetime of the *cis*/*trans* conformers, but not nearly to the same extent as nickel. The addition of DNA to zinc-bound 5F–Trp–HpNikR results in the *cis*/*trans* signature reminiscent of the spectra of the DNA-bound protein in the presence of nickel (Fig. 4*A*). However, the distinct downfield and upfield resonances that characterize the *cis* configuration are more broadened and less separated, indicative of weaker binding compared to the nickel-bound protein, but tighter than that of the apo-protein. Accordingly, fluorescence anisotropy measurements reveal that zinc results in weaker DNA binding to the *ureA* promoter sequence than nickel, consistent with previous findings (Fig. 4*C*) (30), indicating that the metal-induced allosteric response is exquisitely tuned to nickel binding.

## Discussion

Nickel is an essential nutrient for the survival of *H. pylori* in the human stomach (6). HpNikR maintains the balance between nickel starvation and toxicity by activating or repressing the transcription of genes encoding proteins involved in nickel storage, utilization, and transport (17, 19, 23). However, HpNikR can also regulate a variety of other genes, such as those involved in acid adaptation, and is considered a “master regulator” in *H. pylori* (17, 20, 21, 22, 23, 31). In order to enact this regulation, complex formation with specific DNA sequences in the promoters of target genes is allosterically activated when HpNikR binds nickel (27, 28, 29, 30). Previous structural studies indicated that nickel does not induce a large change in tertiary structure (32, 33, 36, 45, 47), but the mechanism by which the metal ion activates DNA binding has until now been unclear. Here, ^19^F-NMR reveals that the apo-protein rapidly samples the *open* and *cis*/*trans* states and that nickel binding shifts the equilibrium toward the DNA-binding competent *cis*/*trans* states, while also increasing the *cis*/*trans* lifetime. In addition, the DNA-bound signature observed in the ^19^F-NMR spectrum of Ni(II)–5F–Trp–HpNikR with the *ureA* promoter is consistent with the *cis* conformation (38, 53).

In a previous study, NMR relaxation measurements performed on the ^13^C/^15^N-labeled DBDs isolated and purified separately from the MBD revealed fast motions of the flexible interdomain linker on a picosecond–nanosecond timescale, as well as fast timescale motions of the regions involved in DNA binding, consistent with our ^19^F-NMR results for the apo-protein (47). The prior NMR studies were performed with triple labeled (^13^C/^15^N/^2^H) full-length protein, but due to the large size of HpNikR (68 kDa), there was extensive signal overlap and broadening, making complete assignment of backbone resonances challenging (45, 47). Nevertheless, while shifts were observed in the ^15^N,^1^H TROSY-HSQC spectra upon binding nickel, it was not possible to interpret this in terms of a shift in equilibrium between an *open* and a *cis*/*trans* state (45).

Analysis of the temperature dependence of the ^19^F-NMR spectra of nickel-bound HpNikR suggests that the formation of the *cis*/*trans* conformer might be enthalpically and entropically driven. Moreover, equivalent measurements in both D_2_O and H_2_O suggest that water-mediated hydrogen bonded networks could play a role in increasing the lifetime of the *cis*/*trans* state. A recent study demonstrated that functional dynamics involving surface water molecules play a role in the allosteric mechanism of the zinc-responsive CzrA metalloregulator from *Staphylococcus aureus* (56). A key feature of the allosteric regulation of DNA binding in that case is a dynamic redistribution between the protein and the surrounding water molecules, which occurs without significant changes to the protein structure upon zinc binding (56). In the case of HpNikR, the observation that the surrounding water molecules could potentially be playing a role in nickel-mediated allosteric activation of DNA binding provides an avenue for future work.

The ^19^F-NMR spectrum of nickel-bound 5F-Trp-HpNikR in complex with the 32 bp *ureA* promoter suggests that the protein binds this promoter in a slightly asymmetric *cis* conformation. This interpretation is supported by the spectrum of the protein bound to a version of the promoter with a perfectly symmetric palindrome (*ureA*–perf) (25). Asymmetric binding to the *ureA* promoter was also observed in a previous study that introduced nuclease activity to HpNikR and subsequently detected asymmetric cleavage patterns when bound to the *ureA* promoter (53). A thymine base present on only one half-site of the pseudo-symmetric palindrome is crucial for high-affinity binding, making it a likely source for the slightly asymmetric conformation adopted by HpNikR (25, 27). Fluorescence anisotropy measurements indicate that HpNikR binds the 32 bp *ureA*-perf promoter with a similar affinity to the unmodified *ureA* promoter; however, previous analysis of the *ureA*–perf sequence demonstrated a slightly weaker affinity compared to the unmodified sequence (*i.e.*, *K*_d_ = 143 ± 10 nM to *ureA*-perf and 67 ± 0.1 nM to *ureA*, respectively using a 49 bp version and fit to a 1:1 binding model) (25). The same group also showed that HpNikR binds a 32 bp version of the *ureA* promoter more tightly than the 49 bp version, with a *K*_d_ of 14 ± 2.9 nM, although these authors did not try a 32 bp version of *ureA*-perf (25). Therefore, the discrepancy of the *ureA*–perf results is likely due to the difference in the length of the sequence. A study using surface plasmon resonance (SPR) spectroscopy revealed that Ni(II)–HpNikR binds to the *ureA* promoter in a two-step process (57), which could be linked to the asymmetric DNA binding. It is therefore likely that the protein binds DNA one domain at a time, possibly through a *trans*-intermediate state. Furthermore, there is evidence that nonspecific contacts between the NikR MBD and DNA contribute to this process (38, 47, 58). This two-step binding mode is likely the reason why the fluorescence anisotropy results fit better to the Hill equation when n≅2, which implies DNA binding is cooperative (Fig. 4*C*, Fig. S1*D*, Fig. S5). These observations overall highlight the importance of the DNA sequence in tuning the activity of HpNikR. This protein has a large regulon, and there is evidence that it can bind promoters with different conformations and affinities (20, 21, 22, 23, 25, 31, 53). The results of these NMR studies support the key role of the DNA sequence in modulating the HpNikR–DNA complexes.

The allosteric response produced by nickel was also compared to the response produced by zinc, a noncognate metal known to bind HpNikR and activate weak DNA binding (this study and (30)). The ^19^F-NMR spectrum of zinc-bound 5F-Trp-HpNikR revealed that although zinc binds, it does not establish a long-lived *cis*/*trans* conformer in the absence of DNA. In addition, the spectrum of Zn(II)–5F–Trp–HpNikR in a complex with the *ureA* promoter indicates weaker binding than in the case of the nickel-activated complex, which is consistent with a study that showed zinc could not induce urease expression *in vivo* (59). It is generally believed that the cognate metal binds to a metalloregulator with a unique coordination in order to activate the correct allosteric response (16, 48, 49, 60, 61). Zinc and nickel ions typically exhibit distinct coordination environments, so it is not surprising that zinc results in a weakened allosteric response upon binding HpNikR (62).

**Figure 5:**
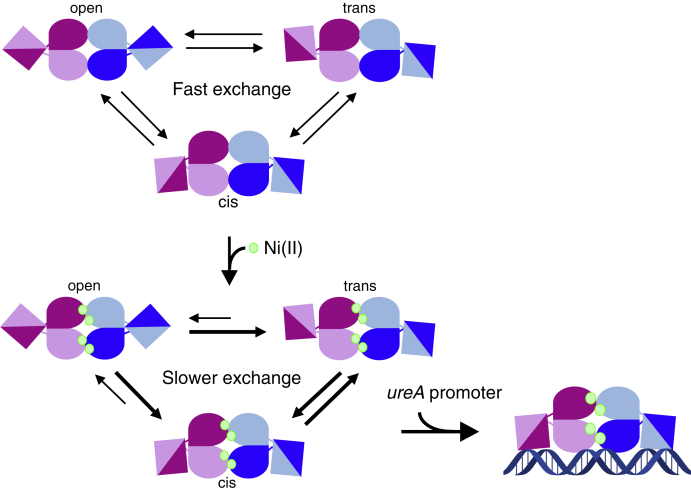
**Schematic of the proposed mechanism of nickel-activated DNA binding by HpNikR.** In the absence of nickel and DNA, the protein is in fast exchange between the different conformational states through fast mobility of the DNA-binding domains. Upon binding nickel, the exchange slows down and the equilibrium is shifted toward the *cis*/*trans* states, which have an increased lifetime. Upon binding the *ureA* promoter, the protein is stabilized in a slightly asymmetric *cis* conformation.

Based on this work, we propose that in the absence of nickel and DNA, HpNikR is best described as an ensemble of rapidly exchanging functional states, which encompass the *open* and *cis*/*trans* conformers. Nickel binding shifts the equilibrium toward the *cis*/*trans* states and, due to reduced DBD mobility, results in slower exchange between the *cis*/*trans* and *open* states. The addition of the *ureA* promoter shifts the equilibrium to a slightly asymmetric DNA-bound *cis* conformation while further reducing the local dynamics in a manner consistent with conformational selection (Fig. 5).

A survey of the allosteric responses of various metalloregulators reveals that they generally fall on a continuum from large conformational changes to subtle changes in dynamics (16). In HpNikR, large changes in dynamics and the conformational equilibrium are observed in response to nickel. Therefore, nickel produces a long-range allosteric effect, which must be propagated to the flexible interdomain linkers or the interdomain interface to influence the mobility of the DBDs and promote DNA binding (16, 36, 45, 46, 47, 63). For some metalloregulators, this allosteric response occurs through a H-bonding network linked directly to the metal-binding residues (16, 64). Furthermore, a redistribution of internal dynamics was recently uncovered as an allosteric response that could be induced by metal binding (16, 65, 66). In particular, it was observed that when the aforementioned CzrA metalloregulator binds zinc, the fast methyl side-chain dynamics that occur throughout the DNA-bound apo-protein are redistributed, thereby quenching a network of conditional motions, leading to inhibition of DNA binding (65). The zinc-responsive AdcR also exhibits a redistribution of protein dynamics upon zinc binding, resulting in quenching of both global and local motions, while also activating fast motions in the DNA-binding motifs to promote DNA binding (66). Future studies will focus on the energetic redistribution that occurs upon nickel binding to HpNikR.

## Experimental procedures

### Protein expression and purification

HpNikR was expressed from the pET24bhpnikRG27 plasmid transformed into BL21(DE3) *E. coli* cells as previously described (67). In brief, cells were grown at 37 °C in 3 litres of LB media containing 50 μg/ml kanamycin and induced at an OD_600_ of 0.6 to 0.9 with 0.3 mM isopropyl-β-D-thiogalactopyranoside (IPTG). After 4 h, the cells were harvested and lysed by sonication in protein buffer (20 mM Tris, pH 7.6, and 100 mM NaCl). The lysate was passed through a 0.45 μm syringe filter and then loaded onto a DEAE Sepharose anion-exchange column (GE Healthcare, Chicago, IL) equilibrated with 20 mM Tris, pH 7.6 and eluted with a linear NaCl gradient. Fractions were screened using 12.5% SDS-PAGE, and those containing HpNikR were pooled and dialyzed overnight against 20 mM Tris, pH 7.6, 1 mM DTT, and 10 mM EDTA at 4 °C. The protein was then loaded onto a MonoQ (GE Healthcare) column for anion-exchange chromatography, initially equilibrated with 20 mM Tris, pH 7.6 and eluted with a linear NaCl gradient. Fractions were again analyzed by 12.5% SDS-PAGE, and those containing HpNikR were collected and stored at 4 °C.

For biosynthetic ^19^F-labeling of HpNikR, pET24bhpnikRG27 was transformed into BL21(DE3) cells. A single colony was selected and used to inoculate 50 ml of M9 minimal media (6.8 g/l Na_2_PO_4_, 3 g/l KH_2_PO_4_, 1 g/l NH_4_Cl, 0.5 g/l NaCl, 10 μg/ml thiamine, 10 μg/ml biotin, 2 mM MgSO_4_, 0.1 mM CaCl_2_, 0.4% glycerol, 50 μg/ml kanamycin) supplemented with 10% LB, and grown overnight at 37 °C. The overnight culture was used to inoculate 1 litre of M9 minimal media. The cells were grown at 37 °C and upon reaching an OD_600_ of 0.6 to 0.9, were incubated with 1 g/l glyphosate (Sigma-Aldrich, St Louis, MO) at 37 °C for 30 min. After a 30 min incubation period, 50 mg each of phenylalanine, tyrosine, and 5-fluorotryptophan (Sigma-Aldrich) was added and immediately induced with 1 mM IPTG for 18 h at 25 °C before harvesting. The ^19^F-labeled proteins were purified as described above.

^19^F-labeled and unlabeled HpNikR concentrations were determined in protein buffer by measuring the electronic absorption at 280 nm and using an extinction coefficient of 8480 M^−1^ cm^−1^ (68). The molecular mass of each protein was confirmed by electrospray ionization mass spectrometry. The observed mass of unlabeled HpNikR was 17,121 Da (predicted 17121.53 Da). ^19^F-labeled HpNikR was 17,139 Da (predicted 17139.53 Da), and electrospray ionization mass spectrometry indicated that the purified protein was consistently >90% ^19^F-labeled (Fig. S1*A*). A 5,5′-dithiobis-(2-nitrobenzoic acid) (DTNB) assay was used to verify that the protein was not oxidized. For the DTNB assay, protein samples and 7 to 56 μM β-mercaptoethanol standards were prepared in 6 M GuHCl, 1 mM EDTA (pH 8.0), and 400 μM DTNB, and the absorbance was measured at 412 nm. The protein was used if it was over 90% reduced.

### Nickel titration

Electronic absorption spectra were collected at 25 °C on an Agilent 8452 spectrophotometer (Santa Clara, CA). Increasing amounts of NiSO_4_ were titrated into a fixed concentration of protein every 10 min at 25 °C, and the absorbance was measured at 302 nm. The difference absorbance spectrum was generated by subtracting the spectrum of the apo-protein from that of the nickel-bound protein. Nickel titrations confirmed that the 5F–Trp–labeled HpNikR is capable of binding stoichiometric nickel (Fig. S1*B*) (28).

### Circular dichroism spectroscopy

Circular dichroism (CD) spectra were recorded on an Olis DSM 1000 CD Spectrophotometer (Bogart, GA) at 20 °C. Protein samples were prepared by diluting to 20 μM and dialyzing overnight against 100 mM potassium phosphate buffer, pH 7.6, at 4 °C. Protein sample concentrations were verified by absorption spectroscopy following collection of the CD spectra. The CD spectra were acquired across 190–260 nm at 1 nm intervals with an integration time of 2 s, and a total of five replicate spectra were collected and averaged to obtain the final spectrum for each sample. The mean residue ellipticity [θ] (deg cm^2^ dmol^−1^) was calculated from the measured ellipticity (θ, mdeg) using equation (1):

$$\left[\theta \right]\;=\;\frac{\theta \;X\;100}{nlc},$$

where *n* is the number of residues, *l* is the path length (cm), and *c* is the protein concentration (mM). The CD spectra of both the apo- and nickel-bound 5F-Trp-HpNikR are similar to those of the unlabeled protein, indicating that fluorine incorporation does not cause substantial changes to the secondary structure (Fig. S1*C*).

### Fluorescence anisotropy assay

The 32 bp fluorescein [F]-labeled and unlabeled oligonucleotides containing the HpNikR recognition sequence from the *ureA* promoter and the modified *ureA*–perf promoter were purchased from Integrated DNA Technologies (IDT; Coralville, IA): *ureA*, 5′-ATATAACACTAATT[F]CATTTTAAATAATAATTA-3′ and 5′-TAATTATTATTTAAAATGAA TTAGTGTTATAT-3′; *ureA*–perf, 5′- ATATATTATTAATT[F]CATTTTAAATAATAATTA-3′ and 5′-TAATTATTATTTAAAATGAATTAATAATATAT-3′ (25). The fluorescein molecule [F] was conjugated to a central thymine located in the spacer between the two half-sites of the HpNikR recognition sequence, where it is unlikely to interfere with HpNikR binding (29). The oligonucleotides were annealed as described previously (29, 36), by combining a 1.25:1 ratio of unlabeled to labeled oligonucleotide in 10 mM Tris, 10 mM NaCl, pH 8.0. The mixture was heated to 85 °C for 5 min on a heating block, which was then immediately turned off allowing the mixture to cool to room temperature. The double-stranded oligonucleotides were stored at −20 °C following quantification by absorption spectroscopy at 260 nm. The extinction coefficient of the DNA duplex (*ε*_D_) was calculated from the extinction coefficients of each strand, $\varepsilon_{S1}$ and $\varepsilon_{S2}$, and hypochromicity (*h*) using equations (2, 3, 69):

$$h\left(260\;nm\right)\;=\;f_{AT}\;\times \;0.287\;+\;f_{GC}\;\times \;0.059,$$

$$\varepsilon_{D}\;=\;\left(1\;-\;h\right)\left(\varepsilon_{S1}\;+\;\varepsilon_{S2}\right),$$

where $f_{AT}$ and $f_{GC}$ are the fractions of AT and GC base pairs, respectively.

The metal-bound proteins used for these experiments were prepared by incubating the protein with 1.2 equivalents of NiSO_4_ or 1.6 equivalents of ZnSO_4_ per protein monomer in protein buffer supplemented with 3 mM MgSO_4_ for 1.5 h at room temperature. Increasing concentrations of nickel-bound protein were then incubated with the fluorescently labeled *ureA* promoter (*ureA*-F) or *ureA*–perf (*ureA*-perf-F) promoter on a black Nunc 384-well plate for a final concentration of 5 nM oligonucleotide. Fluorescence anisotropy measurements were taken on a CLAIROstar Fluorescence Plate Reader (BMG Labtech, Ortenberg, Germany) with an excitation wavelength of 482 nm and an emission wavelength of 540 nm. The data were analyzed by converting the anisotropy, *r*, to fraction bound *F*_bound_ (the fraction of protein bound to DNA at a given DNA concentration), using equation (4, 29, 70):

$$F_{\text{bound}}=\;\frac{r-r_{free}}{\left(r_{\text{bound}}-r\right)Q\;+\;(r-r_{\text{free}}),}$$

where *r*_free_ is the anisotropy of the fluorescein-labeled oligonucleotide, *r*_bound_ is the anisotropy of the DNA–protein complex at saturation, and *Q* is the quantum yield ratio of the bound to free form, calculated from the change in fluorescence intensity (*Q* = *I*_bound_/*I*_free_). *F*_bound_ was plotted against the protein concentration, treating the protein as a tetramer, and fit using the Hill equation (5):

$$F_{\text{bound}}=\;\frac{{\left[P\right]}^{n}}{{K_{d}}^{n}\;+\;{\left[P\right]}^{n}},$$

where *P* is the concentration of protein, *K*_d_ is the protein concentration required for 50% DNA binding, and n is the Hill coefficient. Fluorescence anisotropy experiments indicated that the 5F-labeled and unlabeled proteins have similar DNA-binding affinities for the 32 bp oligonucleotide containing the HpNikR recognition sequence from the *ureA* promoter (Fig. S1*D*) (21, 25, 28, 29, 30, 47).

### Sample preparation for ^19^F-NMR

Samples contained 400 to 900 μM protein monomer in 10% D_2_O, 3 mM MgSO_4_, 20 mM Tris, and 100 mM NaCl and were buffered to pH 7.6. Samples containing metal were preincubated overnight at 4 °C with 1.2 equivalents of NiSO_4_ or 1.6 equivalents of ZnSO_4_ per protein monomer. The oligonucleotides used were HPLC-purified and purchased from IDT as a duplex: *ureA*, 5′-ATATAACACTAATTCATTTTAAATAATAATTA-3′; *ureA*–perf, 5′-ATATATTATTAATTCATTTTAAATAATAATTA-3′. The amount of oligonucleotide added was equal to the tetrameric protein concentration, unless stated otherwise. Samples with excess DNA were incubated with 1.5 equivalents to the tetrameric protein concentration. For solvent-induced isotopic shift measurements, the buffer was exchanged from 90% H_2_O to ∼90% D_2_O by diluting the sample 1:10 with deuterated protein buffer (20 mM Tris 100 mM NaCl, pH 7.6; made by dissolving Tris and NaCl in D_2_O and then adjusting the pH) (71), followed by concentration using Amicon Ultra 3 kDa molecular weight cutoff centrifugal filters (Millipore, Burlington, MA), and repeating three times. After exchanging the buffer, 3 mM MgSO_4_ was added to the sample. Each sample also included 100 μM p-fluorophenylalanine (Sigma-Aldrich) to produce a reference peak to which the spectra were aligned.

### ^19^F-NMR experiments

All NMR spectra were acquired at 25 °C, unless stated otherwise, on a 700 MHz Agilent DD2 spectrometer equipped with a HFX probe. Free induction decay signals were acquired with a π/2 pulse length of 8 μs, a repetition time of 1 s, a spectral with of 33 kHz, and an acquisition time of 1.25 s. Most spectra were acquired with 8000 scans, except those originating from samples containing DNA, which were acquired with 18,000 to 20,000 scans, depending on the sample concentration. The free induction decay was apodized with a Lorentzian filter equivalent to 10 Hz line broadening. All spectral processing was performed using Mnova 12.0.0 (Mestrelab Research).

^19^F transverse relaxation times (*T*_2_) were determined at 10 °C using a Carr–Purcell–Meiboom–Gill (CPMG) *T*_2_ pulse sequence, using a refocusing period of 1 ms, with a total transverse magnetization evolution time, *T*, of 0.4, 0.8, 1.2, and 1.7 ms. *T*_2_ relaxation times were calculated by fitting plots of the ^19^F signal intensity (*I*) *versus* transverse magnetization evolution times to equation (6):

$$I\left(T\right)\;=\;I\left(0\right)e^{-T/T_{2}}$$

The contribution to the linewidth originating from homogeneous (dynamic) processes was estimated from *T*_2_ relaxation times using equation (7):

$$T_{2}\;=\;\frac{1}{\pi \;\times \;\text{linewidth}}$$

### Van’t Hoff analysis

Using the deconvolved spectra of the nickel-bound protein, the peak areas of the resonances corresponding to the different states (*S*_*cis/trans*_ and *S*_*open*_) were interpreted as relative populations (*P*_*cis/trans*_ and *P*_*open*_) (72). Assuming that state-specific enthalpies and entropies are constant over the investigated temperature range, the temperature dependence of the corresponding equilibrium constant *K*_*open,cis/trans*_ (=*P*_*cis/trans*_*/P*_*open*_) may be expressed by the van’t Hoff equation (8),

$$\ln\;K_{open,cis/trans}\;=\;-\frac{\Delta H_{open,cis/trans}^{{}^{\circ}}}{RT}+\frac{\Delta S_{open,cis/trans}^{{}^{\circ}}}{R},$$

where $\Delta H_{open,cis/trans}^{{}^{\circ}}$ and $\Delta S_{open,cis/trans}^{{}^{\circ}}$ represent the standard enthalpy and entropy differences between the states, *R* is the gas constant, and *T* is the absolute temperature.

## Data availability

All data are contained within the article and the supporting information.

## Conflict of interest

The authors declare that they have no conflicts of interest with the contents of this article.
