# Supplementary material for: Allosteric regulation of the nickel-responsive NikR transcription factor from Helicobacter pylori
Source: J Biol Chem. 2020 Nov 22;296:100069. doi: 10.1074/jbc.RA120.015459 (PMC7949043; doi:10.1074/jbc.RA120.015459)
Supplement: Supplementary Figures and Table [file mmc1.pdf]

Supporting Information for:

Allosteric regulation of the nickel-responsive NikR transcription factor from *Helicobacter pylori*

Karina A. Baksh[1], Dmitry Pichugin[2], Robert Scott Prosser[1,2,*], and Deborah B. Zamble[1,2]

[1]Department of Biochemistry, University of Toronto, Toronto, Ontario, Canada M5S 1A8
[2]Department of Chemistry, University of Toronto, Toronto, Ontario, Canada M5S 3H6

This supporting information includes:

Figure S1. Characterization of 5F-Trp labeled HpNikR compared to unlabeled HpNikR

Figure S2. ^{19}F-NMR T_2 relaxation profile of apo-5F-Trp-HpNikR deconvolved into one resonance

Figure S3. ^{19}F-NMR T_2 relaxation profile of Ni(II)-5F-Trp-HpNikR deconvolved into two or three resonances

Table S1. Comparison of linewidths extracted from T_2 relaxation experiments and deconvolutions of Ni(II)-5F-Trp HpNikR

Figure S4. Deconvolved ^{19}F-NMR spectra of Ni(II)-5F-Trp-HpNikR-DNA with one equivalent of DNA at 25°C and 35°C, and 1.5 equivalents of DNA at 25°C

Figure S5. Fluorescence anisotropy-monitored binding of apo- and Ni(II)-HpNikR with the *ureA* and *ureA*-perf promoters.

Figure S6. Change in ^{19}F chemical shift of apo- and nickel-bound 5F-Trp-HpNikR in 10% versus ~90% D_2O

Figure S7. Van't Hoff analysis of Ni(II)-5F-Trp-HpNikR in 10% versus ~90% D_2O

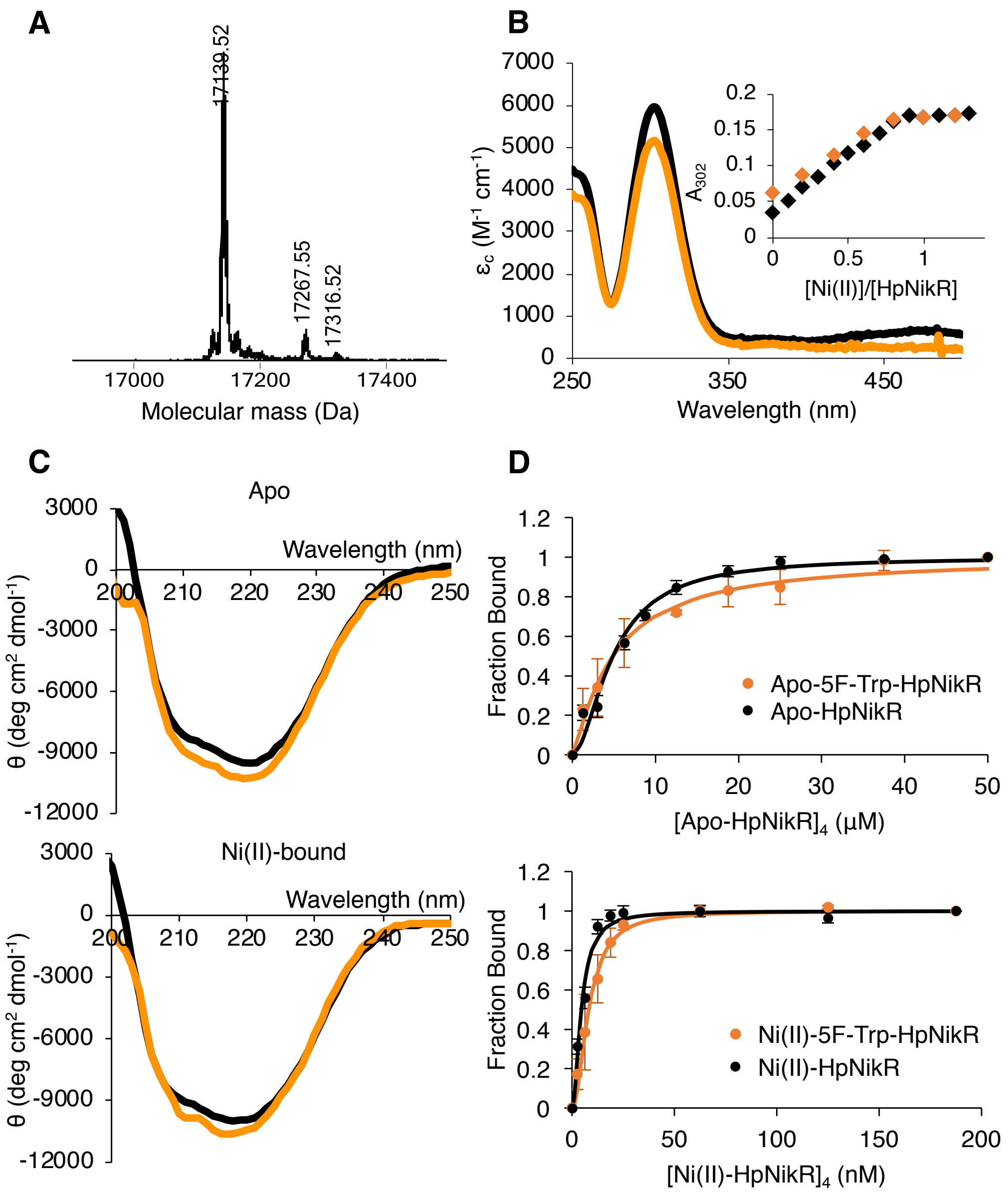

Figure S1. (A) ESI-MS spectrum of 5F-Trp-HpNikR. The mass matches the theoretical molecular mass of 17139 Da. (B) The difference absorption spectrum of nickel bound to unlabeled (black) and 5F-Trp-labeled (orange) HpNikR. The peak at 302 nm represents a ligand-to-metal charge transfer from the thiol group of cysteine to nickel. Inset: titration of HpNikR (black) and 5F-Trp-HpNikR (orange) with nickel produces a linear increase in absorbance at 302 nm until saturation near 1 equivalent of nickel. (C) Circular dichroism spectra of apo- and nickel-bound unlabeled (black) and 5F-Trp-labeled (orange) HpNikR indicate similar secondary structures. (D) Fluorescence anisotropy monitored binding of nickel-bound unlabeled (black) and 5F-Trp-labeled (orange) HpNikR with 5 nM of the *ureA*-F promoter. The data points represent the average derived from the preparation of three samples at each protein concentration, and the error bars represent ± one standard deviation. The data from each replicate were fit to the Hill equation. The average DNA binding affinities for the apo unlabeled and 5F-Trp-labeled proteins were determined to be 4.9 ± 1.7 μM, n = 1.8 ± 0.4 and 4.9 ± 0.4 μM, n = 1.2 ± 0.2, respectively. The average DNA binding affinities for the nickel-bound unlabeled and 5F-Trp-labeled proteins were determined to be 4.5 ± 0.6 nM, n = 1.8 ± 0.1 and 8.1 ± 3 nM, n = 1.9 ± 0.4, respectively. The uncertainties represent the standard deviation of the K_d values calculated from the three replicates.

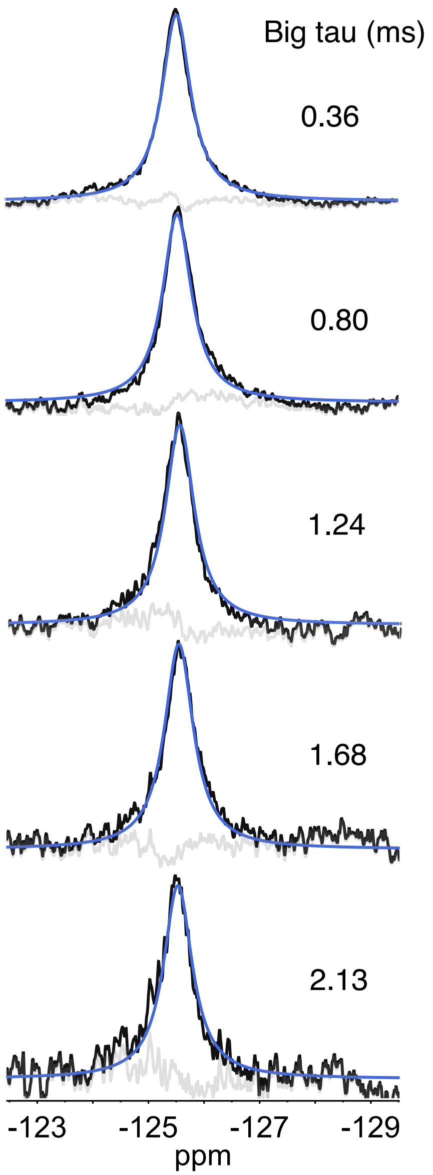

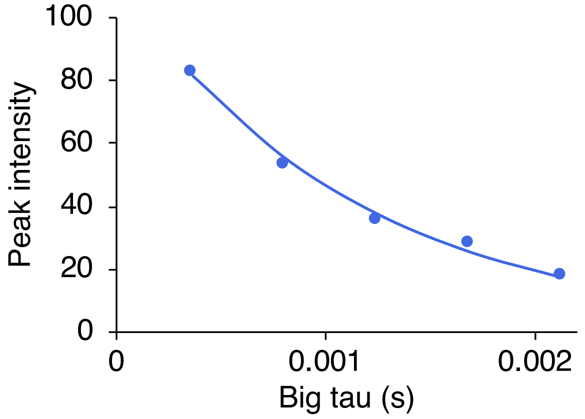

T_2 (ms)	1.1 ± 0.2
Linewidth from T_2 (Hz)	279
Linewidth from line fitting (Hz)	328
Difference (Hz)	49

Figure S2. ^{19}F-NMR T_2 relaxation profile of apo-5F-Trp-HpNikR at 10°C deconvolved into one resonance. Here, "Big tau" represents the total evolution time of transverse magnetization. The blue line corresponds to the fit peak, the faint purple line is the sum of the fits, and the grey line is the residual error associated with the fit. The first spectrum in the relaxation series was deconvolved to estimate the frequency and line width associated with the signal, and for the remaining spectra the only fitting parameter that is changed is the intensity. The decay profile of the signal intensity (right) was used to estimate T_2, and the resulting values are shown in the table.

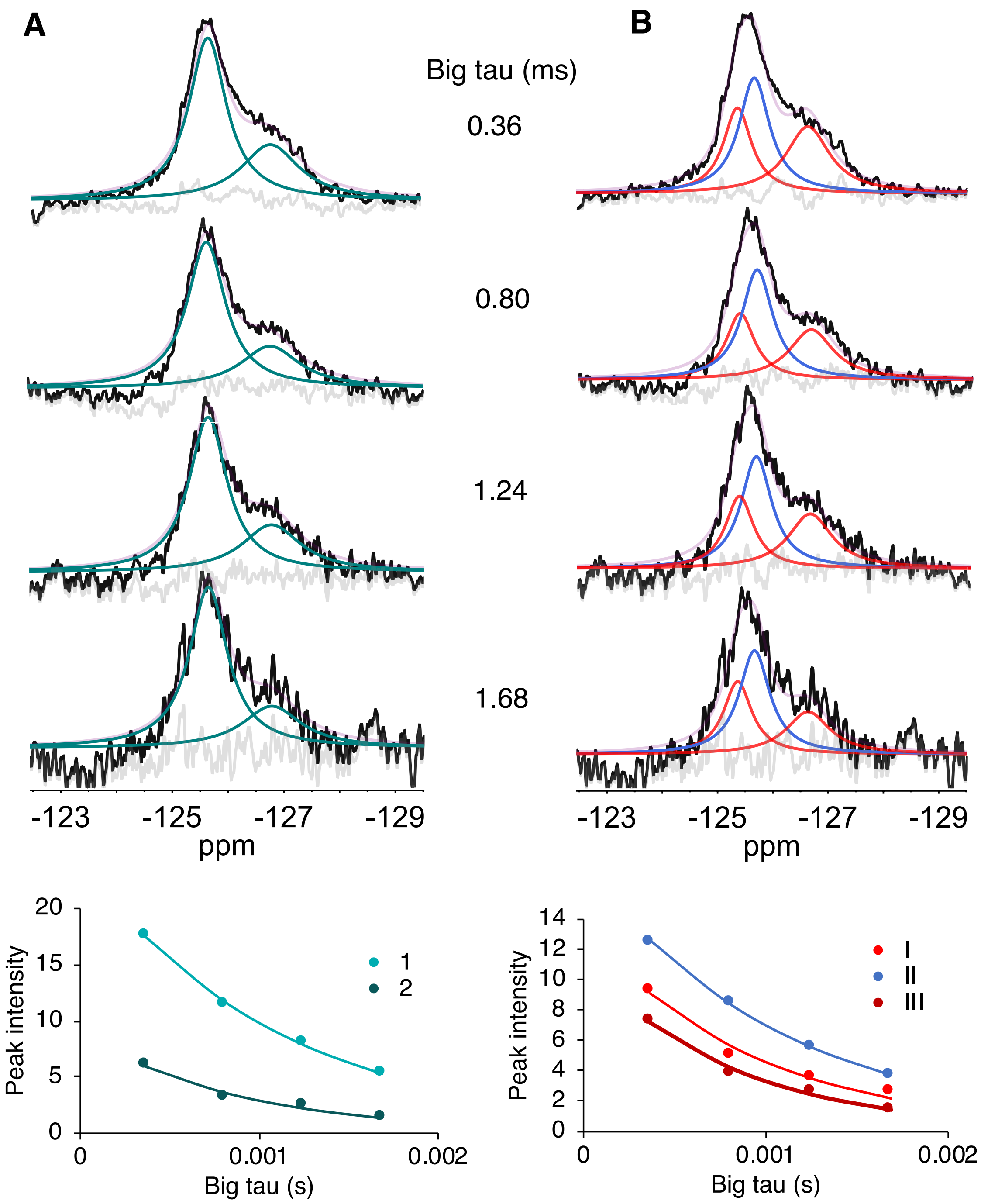

Figure S3. ^{19}F-NMR T_2 relaxation profile of Ni(II)-5F-Trp-HpNikR at 10°C deconvolved into (A) two and (B) three resonances. Here, "Big tau" represents the total evolution time of transverse magnetization. The faint purple line is the sum of the fits, and the grey line is the residual error associated with the fit. The first spectrum in the relaxation series was deconvolved to estimate the frequencies and line widths associated with each signal, and for the remaining spectra the only fitting parameter that is changed are the respective intensities. The decay profiles of the signal intensities (shown below the spectra) were used to estimate T_2 and the resulting values are shown in Table S1.

Table S1. Estimates of linewidths in the spectra of Ni(II)-5F-Trp-HpNikR. The linewidths obtained from deconvolution into two resonances are larger than those obtained from deconvolution into three resonances. The difference in the linewidth of resonance III obtained from T_2 compared to line fitting is large but there is no evidence to suggest the presence of additional resonances within resonance III.

	2 resonances		3 resonances		
	1	2	I	II	III
T_2 (ms)	1.1 ± 0.2	0.89 ± 0.3	0.93 ± 0.4	1.08 ± 0.04	0.83 ± 0.4
Linewidth from T_2 (Hz)	288	357	343	294	385.5
Linewidth from line fitting (Hz)	546	844	407	443	631
Difference (Hz)	257.7	486.7	63.8	148.8	245.5

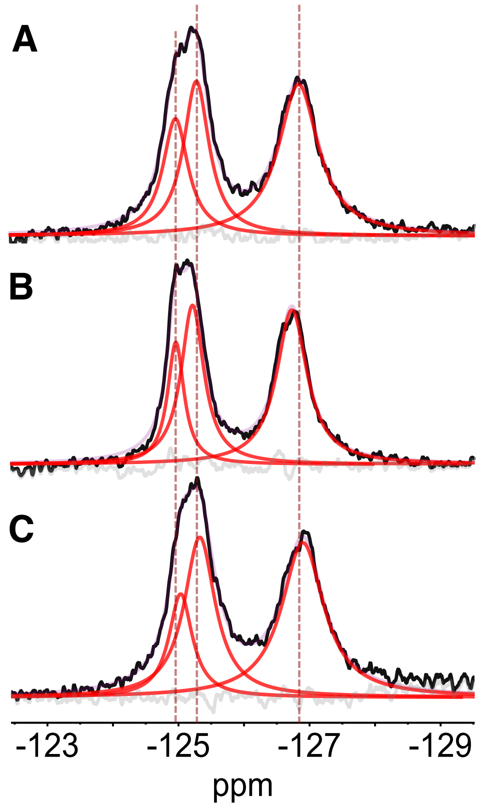

Figure S4. Deconvolved ^{19}F-NMR spectra of Ni(II)-5F-Trp-HpNikR-DNA with one equivalent of the *ureA* promoter at (A) 25°C and (B) 35°C. (C) Spectra of Ni(II)-5F-Trp-HpNikR-DNA with 1.5 equivalents of the *ureA* promoter at 25°C. Fit peaks in red correspond to DNA bound populations, the faint purple line is the sum of the fits, and the grey line is the residual error associated with the fit.

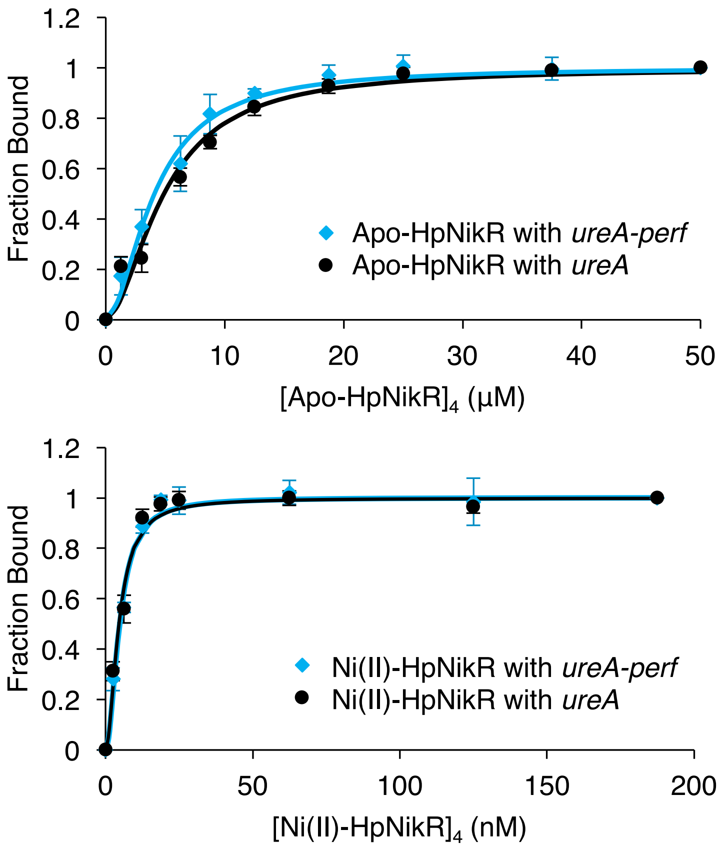

Figure S5. Fluorescence anisotropy monitored binding of (top) apo- and (bottom) nickel-bound HpNikR with 5 nM of the *ureA*-F (black) and *ureA*-perf-F (light blue) promoters. The data points represent the average derived from the preparation of three samples at each protein concentration, and the error bars represent ± one standard deviation. The data from each replicate were fit to the Hill equation. The average DNA binding affinities for the apo-protein were determined to be 4.9 ± 1.7 μM, n = 1.8 ± 0.4 and 4.1 ± 1.1 μM, n = 1.8 ± 0.2 for the *ureA* and *ureA*-perf promoters, respectively. For the nickel-bound protein, the average DNA binding affinities were determined to be 4.5 ± 0.6 nM, n = 1.8 ± 0.1 and 4.7 ± 0.4 μM, n = 1.9 ± 0.2 for the *ureA* and *ureA*-perf promoters, respectively. The uncertainties represent the standard deviation of the K_d values calculated from the three replicates.

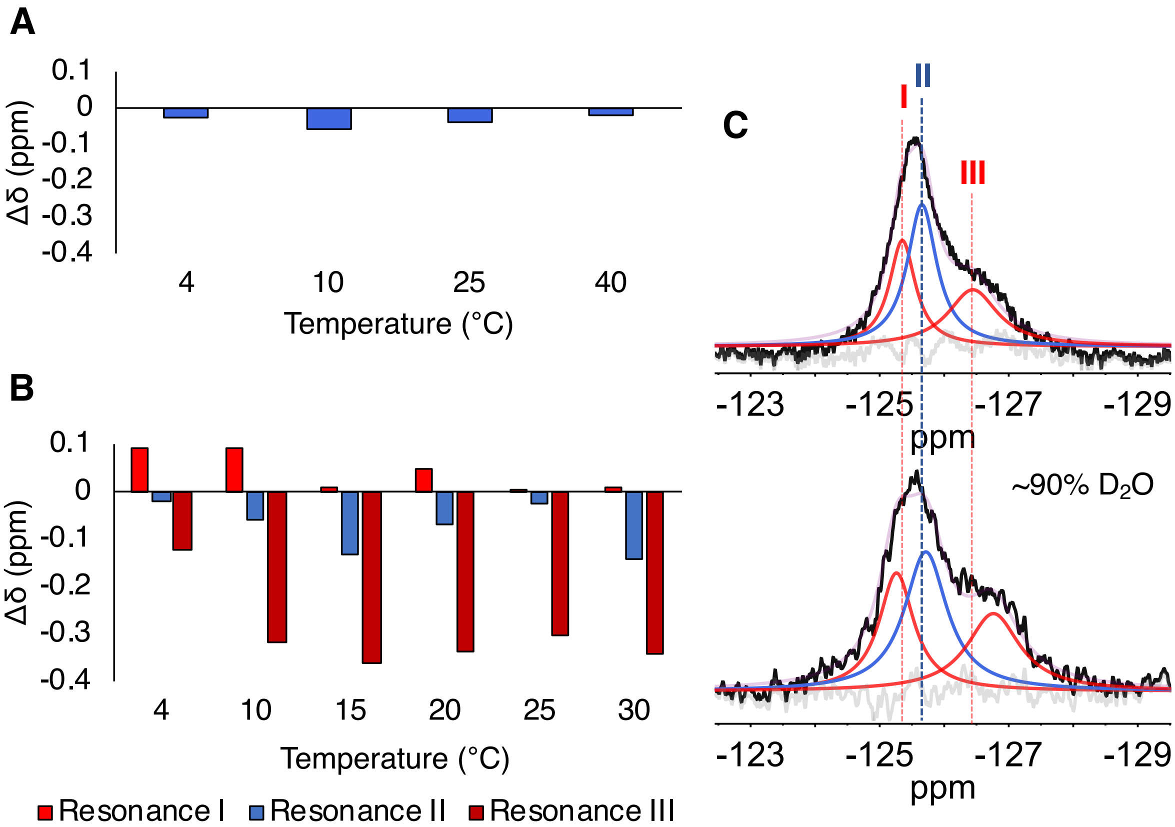

Figure S6. (A) Change in ^{19}F chemical shift of apo-5F-Trp-HpNikR in 10% versus ~90% D_2O ($\Delta\delta = \delta_{D2O} - \delta_{H2O}$). (B) Change in ^{19}F chemical shift of nickel-bound 5F-Trp-HpNikR in 10% versus ~90% D_2O when deconvolved into three resonances. The alternative deconvolution into two resonances is not shown. Values were calculated from deconvolutions of the spectra shown in Figure 3A and B. (C) Deconvolved ^{19}F-NMR spectra of Ni(II)-5F-Trp-HpNikR in 10% (top) and ~90% D_2O (bottom) at 10°C to show the shift of each resonance.

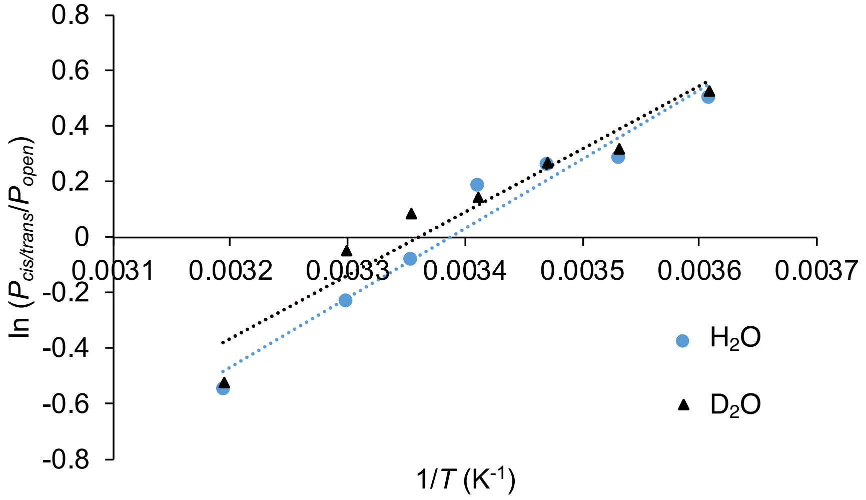

Figure S7. Van't Hoff analysis of the equilibrium constant $K_{open,cis/trans}$ for the nickel-bound 5F-Trp-HpNikR.
